# Incidence of Trigger Finger in Surgically and Nonsurgically Managed Carpal Tunnel Syndrome

**DOI:** 10.1016/j.jhsg.2022.10.017

**Published:** 2022-11-24

**Authors:** Lauren E. Wessel, Alex Gu, Paul Asadourian, Jeffrey G. Stepan, Duretti T. Fufa, Daniel A. Osei

**Affiliations:** ∗ Department of Orthopedic Surgery, University of California Los Angeles, Santa Monica, CA; † Department of Orthopedic Surgery, The George Washington University School of Medicine and Health Sciences, Washington, DC; ‡ Columbia University Vagelos College of Physicians and Surgeons, New York, NY; § Department of Orthopedic Surgery, The University of Chicago Medicine, Chicago, IL; ‖ Department of Hand Surgery, Hospital for Special Surgery, New York, NY

**Keywords:** Epidemiology, Carpal tunnel release, Carpal tunnel syndrome, Trigger finger

## Abstract

**Purpose:**

The purpose of this study was to determine whether extremities undergoing carpal tunnel release (CTR) have an increased rate of trigger finger (TF) compared with conservatively managed carpal tunnel syndrome.

**Methods:**

Data were collected from the Humana Insurance Database, and subjects were chosen on the basis of a history of CTR with propensity matching performed to develop a nonsurgical cohort. Following propensity matching, 16,768 patients were identified and equally split between surgical and nonsurgical treatments. Demographic information and medical comorbidities were recorded. Univariate and multivariate analyses were performed to identify risk factors for the development of TF within 6 months of carpal tunnel syndrome diagnosis.

**Results:**

Patients in the surgical cohort were more likely to develop TF than those in the nonsurgical cohort whether in the ipsilateral or contralateral extremity. Whether managed surgically or nonsurgically, extremities with carpal tunnel syndrome demonstrated an increased prevalence of TF than their contralateral, unaffected extremity.

**Conclusions:**

Surgeons should be aware of the association of TF and CTR both during the presurgical and postsurgical evaluations as they might impact patient management. With knowledge of these data, surgeons may be more attuned to detecting an early TF during the postsurgical period and offer more aggressive treatment of TF pathology during CTR.

**Type of study/level of evidence:**

Prognostic III.

There is a known relationship between carpal tunnel syndrome (CTS) and trigger finger (TF) or stenosing flexor tenosynovitis.^1^, ^2^, ^3^, ^4^, ^5^, ^6^ Previous studies have demonstrated an 11% to 40% coincidence of CTS and TF.^1^^,^^7^, ^8^, ^9^ However, whether one pathology precedes the other is unclear, and studies are further divided as to whether surgical treatment of CTS further increases the risk of TF development.^1^^,^^3^^,^^6^^,^^7^ Additionally, some surgeons believe that the increased postsurgical swelling from carpal tunnel release (CTR) may contribute to a heightened inflammatory cascade and the development of TF.^1^^,^^10^ Specifically, Lin et al^10^ noted that this risk was specifically increased in the first 6 months after carpal tunnel surgery and subsequently stabilized.

The contribution of surgical intervention to the risk of TF development is of interest in providing presurgical counseling and its possible impact on management, such as whether to offer concomitant surgical release or injection of an early TF. Prior studies on this topic have been performed at single centers, where there may be limited practice variation.^1^^,^^3^^,^^6^ To address this limitation, our study used a large administrative database to gather epidemiologic information regarding the development of TF after CTR.

Specifically, among a population of patients with diagnosed CTS, our primary aim was to determine whether extremities undergoing CTR had an increased rate of TF than conservatively managed CTS in a propensity-matched population. Our secondary aims were the following: (1) to determine whether CTR is associated with an increased rate of ipsilateral TF compared with the contralateral extremity, acting as an internal control, and (2) to determine the time course of TF development after CTR. We hypothesized that the extremities that undergo CTR for CTS would show an increased risk of developing TF during a 6-month postsurgical period compared with a nonsurgical cohort.

## Materials and Methods

Data were collected from the Humana Insurance Database using the PearlDiver Patient Records Database^11^ from 2015 to 2017. The PearlDiver database contains records for more than 22 million patients, further describing hospital and physician billing records and procedural information. Subjects with CTS were identified using the International Statistical Classification of Diseases, Tenth Revision (ICD-10) codes. To maintain laterality, only ICD-10 codes were used. Relevant diagnostic and procedural codes are referenced in Appendix 1 (available on the *Journal*’s website at www.jhsgo.org). Demographic information and medical comorbidities were recorded. Using laterality-specific coding, the incidence of the TF diagnosis within 6 months of CTR was determined using the ICD-10 diagnosis codes. The incidence of TF diagnosis among the nonsurgical group was determined within 6 months of CTS diagnosis. The incidence of TF was determined by the concomitant, laterality-specific ICD-10 code and the Current Procedure T erminology code for TF injection or release, indicating a clinically relevant diagnosis of TF. The International Statistical Classification of Diseases, Tenth Revision diagnosis codes associated with unspecified limbs were excluded. Carpal tunnel release was determined on the basis of the presence of a CTR-related Current Procedure Terminology code (Appendix 1).

**Figure 1:**
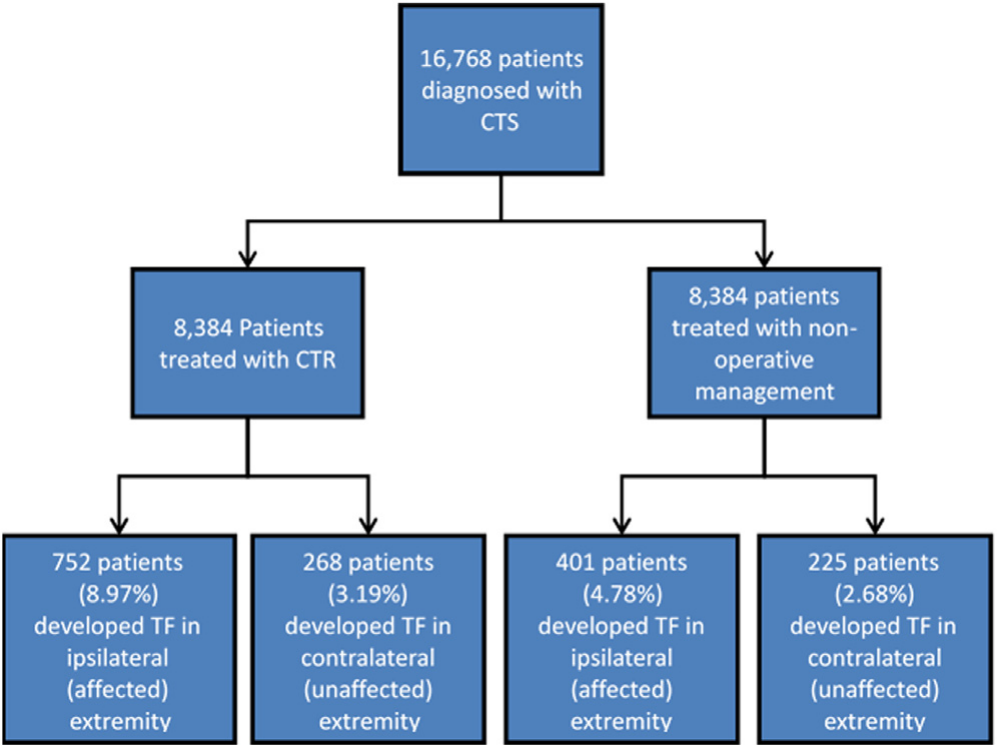
The study population. CTR: Carpal Tunnel Release; CTS: Carpal Tunnel Syndrome; TF: Trigger Finger

Two patient cohorts were identified as follows: (1) surgical cohort: patients who underwent CTR for CTS, and (2) nonsurgical cohort: patients who were treated conservatively for CTS within our study period (Fig. 1). Patients were followed up for the development of TF in the ipsilateral or contralateral extremity. These 2 sets of breakpoints served as a proxy for the severity of disease during the study period (surgical vs nonsurgical management) and controlled for patients’ biology and the effects of surgery (comparison of ipsilateral or contralateral). Patients were required to have a continuous, active status in the database for inclusion in the study to ensure no loss of patients due to changes in insurance carrier status. Additionally, each patient’s contralateral extremity was used as an internal control for each of these cohorts. Patients were excluded if the CTS diagnosis existed bilaterally to maintain a non-CTS internal control for comparison. Additionally, patients were excluded if they had a prior history of TF as determined by ICD-10 attribution or CTS within the lifespan of the database, if laterality could not be determined, or if they had less than 6 months of follow-up. The control of comorbidities was conducted using the Charlson Comorbidity Index (CCI) and was used for propensity matching.^12^

### Propensity score matching

To balance measured and unmeasured covariates, and thus, mitigate potential confounders, we used propensity score matching to create matched cohorts of surgical and nonsurgical patients with CTS. The propensity score was defined as the conditional probability of having undergone CTR based on age, sex, CCI, and diabetes mellitus status. These factors were chosen given their association with the development of TF.^13^^,^^14^ Matching was conducted using a 1:1 nearest neighbor matching by univariate analysis, with the caliper set at 0.02 of the standard deviation. Matching was conducted using demographics collected at the time of CTS diagnosis among the surgical and nonsurgical cohorts. Propensity score matching was conducted using R software provided by PearlDiver.

### Statistical analysis

Data on the patients’ demographics, CCI, history of diabetes mellitus, and incidence of TF were analyzed using univariate and multivariate analyses on the R software provided within PearlDiver. Propensity matching was conducted to reduce potential confounders and provide a more homogeneous cohort. Univariate analysis was first performed using Pearson chi-square or analysis of variance. For the multivariate analyses, logistic regression analyses were performed to determine adjusted associations of risk factors for postsurgical TF. The results were reported as odds ratios (ORs) and 95% confidence intervals (CIs). A *P* value of <.05 was used as the cutoff for significance.

## Results

**Figure 2:**
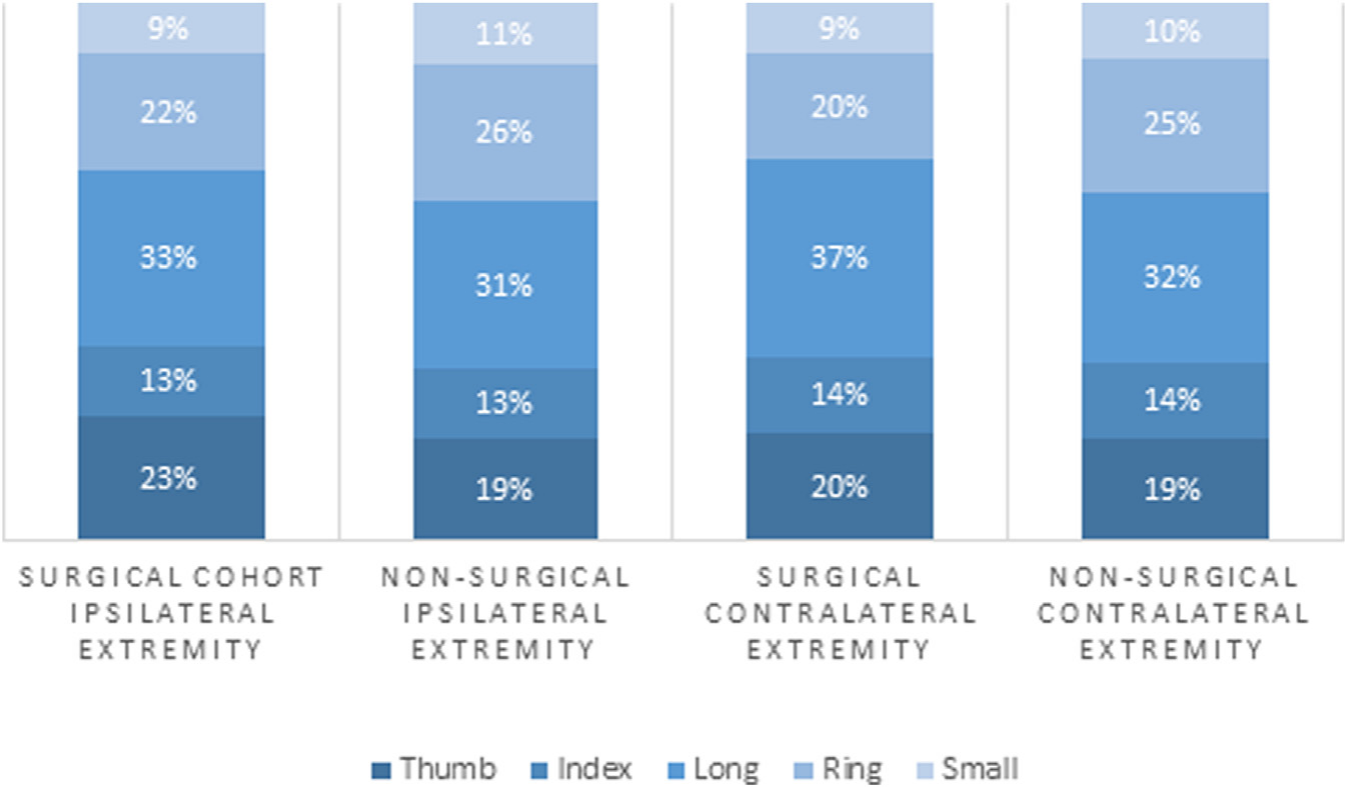
The distribution of trigger finger by cohort.

Following propensity matching, 16,768 patients with a unilateral CTS diagnosis were selected for the study. Among those, 8,384 patients who underwent CTR constituted the surgical cohort and 8,384 patients who underwent conservative management for CTS were selected as their closest propensity-matched controls. Baseline patient demographics and clinical characteristics are listed in the Table. In general, there were no differences between the surgical and nonsurgical cohorts with respect to gender, age, CCI, or diabetes mellitus status (Table). Among all extremities that developed TF, the middle finger was the most common digit affected, followed by the ring and thumb fingers (Fig. 2).

**Table:** Demographics and Medical Comorbidities of Patients Diagnosed With Carpal Tunnel Syndrome

Category	CTR		Non-CTR		*P* Value∗
	N = 8,384		N = 8,384		
	n	%	n	%	
Sex					.823
Male	3,312	39.5	3,310	39.5	
Female	5,072	60.5	5,074	60.5	
Age (y)					.453
<50	564	6.7	566	6.8	
50–59	1,082	12.9	1,080	12.9	
60–69	2,257	26.9	2,258	26.9	
70–79	3,024	36.1	3,029	36.1	
80–89	1,287	15.4	1,286	15.3	
>90	170	2.0	165	2.0	
CCI					.754
0	2,906	34.7	2,913	34.7	
1	1,589	19.0	1,585	18.9	
2	1,073	12.8	1,088	13.0	
3	903	10.8	890	10.6	
4+	1,913	22.8	1,908	22.8	
Diabetes mellitus	664	7.9	673	8.0	.542

∗ Significant *P* values are *P* < .05.

### Trigger finger development among surgical versus nonsurgical cohorts (propensity matching)

In total, 752 (9.0%) extremities in the surgical cohort and 401 (4.9%) extremities in the nonsurgical cohort were treated for TF during the 6-month study period (OR: 1.9; 95% CI: 1.4–2.2; *P* < .001; Fig. 1). There was increased treatment of the thumb (23% vs 19%; *P* < .05) and the middle (33% vs 31%; *P* < .05) finger in the surgical cohort compared with the nonsurgical cohort (Fig. 2). The average time between CTS diagnosis to TF treatment was approximately 8 weeks and was similar between the surgical and conservatively managed cohorts (52.3 vs 56.2 days, respectively, *P* = .102). The overall time from incidence of CTR to ipsilateral TF injection or first annular pulley release was 36.3 days.

### Trigger Finger development versus the contralateral extremity in the surgical cohort (internal control)

Among patients with unilateral CTS who were treated with surgical intervention, 752 (9.0%) ipsilateral extremities and 268 (3.2%) contralateral extremities were treated for TF during the 6-month study period (OR: 2.8; 95% CI: 2.4–3.2; *P* < .01; Fig. 1). There was increased treatment of the thumb (23% vs 20%; *P* < .05) and decreased treatment of the middle (37% vs 33%; *P <* .05) finger in the ipsilateral cohort compared with the contralateral cohort (Fig. 2).

### Trigger finger development versus the contralateral extremity in the nonsurgical cohort (internal control)

Patients who were diagnosed with CTS and treated conservatively also had an increased rate of TF of the ipsilateral compared with the contralateral side. Among 401 ipsilateral extremities, 4.8% were treated for TF during the 6-month study, although there were only 225 (2.7%) contralateral extremities that were treated for TF in the same period (OR: 1.8; 95% CI: 1.4–2.2; *P* = .025; Fig. 1). There was no difference in the frequency of TF between various fingers in the ipsilateral extremity compared with the contralateral extremity.

## Discussion

The relationship between CTS and TF has been investigated in recent studies in the hand literature.^1^, ^2^, ^3^, ^4^, ^5^, ^6^, ^7^^,^^10^^,^^15^, ^16^, ^17^ Yet, the literature does not clearly address whether an association exists between surgical intervention for CTS and the development of TF. We used the recent switch to ICD-10 coding, and thus, the introduction of laterality-specific coding to study a large administrative database of patients diagnosed with CTS. We studied the rates of TF in the CTS-affected extremities and compared the surgical and nonsurgical CTS cohorts. Additionally, we studied the rate of TF development against the respective contralateral extremities of each cohort.

In our propensity-matched cohort analysis, we observed an association between surgical treatment for CTS and the increased rate of development of TF in the ipsilateral extremity compared with extremities with CTS that were managed nonsurgically. In both groups, the development of TF was similar at 8 weeks. In the internal control portion of the study, extremities with CTS diagnosis, irrespective of conservative versus surgical management, demonstrated an increased likelihood of TF development compared with the contralateral upper extremity. As a result, we caution against using these findings to suggest that CTR causes TF. However, they further illuminate the relationship between CTS and TF to raise the level of scrutiny for TF in patients with CTS and counsel patients.

Trigger finger occurs at higher rates in extremities affected by CTS, rheumatoid arthritis, and hypothyroidism, supporting the belief that an inflammatory process may play a role in the development of TF.^18^, ^19^, ^20^ Moreover, a recent retrospective study by Zhang et al^6^ showed a lateral association of CTR and TF, as new-onset TF is 2.5 times more likely to develop in the surgical hand than the contralateral nonsurgical hand within the first postsurgical year. These findings are similar to our reported OR of 2.8 for this cohort. Given the co-occurrence of TF and the systemic inflammatory processes as well as postsurgical states,^21^ we hypothesized that patients who underwent surgical management for CTS would exhibit higher rates of TF than for those managed conservatively.

Prior data on this association in the literature is varied. Although Zhang et al^6^ showed a lateral association between TF and CTR, they did not find a temporal association between CTR and the development of postsurgical TF in their single-center study of retrospectively collected data. Their data indicated that new-onset TF was 50% less likely to develop in the surgical hand during the postsurgical year than the year before CTR. Conversely, many studies have highlighted the presence of the temporal course of postsurgical TF development.^7^^,^^10^^,^^15^^,^^17^ Furthermore, Lee et al^17^ studied the anatomic underpinnings behind this relationship. The authors used ultrasound to determine that patients who developed TF after CTR tended to have significantly increased volar migration of their flexor tendons relative to those who did not develop TF. They hypothesized that this volar migration was made possible by CTR. Our data regarding the comparison of a propensity-matched cohort and the comparison to the contralateral upper extremity support the association between CTR and the development of TF. However, based on these data, we could not determine the cause of the association. Whether this association is secondary to the increased severity of CTS pathology or surgical release remains uncertain.

In our analysis, we determined that the most commonly affected digit in TF following CTR was the middle digit, followed by the ring and thumb digits (Fig. 2). This finding is distinct from the current literature, which most commonly cites the thumb as having the greatest involvement in the setting of CTS.^1^^,^^3^^,^^6^^,^^17^ However, our data support an increase in the involvement of the thumb and the middle digit in the surgical cohort compared with their nonsurgical counterparts, which may account for some discrepancies with prior literature. Additionally, the predilection for median nerve-innervated fingers may indicate that patients had early symptoms in these digits, which may have been masked or underappreciated because of concomitant median neuropathy. This point further underscores the importance of attention to this association in the presurgical patient physical examination and counseling, particularly in that increased inflammation in the postsurgical state may further contribute to the development of CTS.

Regarding the timing of TF onset after CTS diagnosis, we found no difference between the time from CTS diagnosis to TF development between patients whose CTS was managed surgically versus those who underwent conservative management. These data are similar to those published by Zhang et al^6^, which failed to demonstrate a temporal relationship between CTR and new-onset TF. However, whereas Zhang et al^6^ used the period before CTR as a control for TF development, our study used a propensity-matched control cohort of conservatively managed patients with CTS to understand this difference. We believe that our comparison with the ipsilateral upper extremity and a cohort of propensity-matched patients with conservatively managed CTS appropriately controls for the natural history of TF development in our cohort.

This study has several limitations. First, as this retrospective study used Humana insurance records, the study is subject to inaccuracies during the billing process. To limit the impact of these inaccuracies, we eliminated subjects with incomplete or unspecified billing codes and those with less than 6 months of follow-up within the insurance database. Our use of procedure codes in combination with ICD-10 diagnosis of TF focuses the study on clinically relevant TF for which intervention is sought and ensures closer alignment with coding specificity, as providers may be inconsistent in applying ICD-10 codes but are much more accurate at coding in combination with procedural codes. Additionally, our results showing increased TF in CTS extremities treated surgically may be biased by patients treated surgically who were under the care of a surgeon whose experience and threshold to treat TF may have been different than those patients treated nonsurgically and may have been in the care of generalists or specialists. We used a follow-up period of 6 months to determine TF development, which may exclude patients who received treatment for TF outside of the 6-month window. However, we believed that the 6-month follow-up period highlighted cases of TF development associated with CTS diagnosis and that a longer follow-up timeframe may reflect the natural history of TF development. This is reflected in our analysis in that TF treatment, on average, occurs within 2 months of CTS diagnosis in each group. Given the large administrative database, we could not standardize the indications for intervention for CTS or TF. Therefore, heterogeneity in the severity of pathology for any specific intervention may exist within our cohort.

Additionally, given that the purpose of this study was to examine the development of a primary TF in the setting of CTS, we cannot comment on the frequency of recurrent or worsening TF. This may be an area of interest for future study. Lastly, we were unable to determine causality with this administrative database. We acknowledge and even hypothesize that the detected differences between the surgical and nonsurgical cohorts might be secondary to the postsurgical state, a generalized increased inflammatory state, or secondary to more extreme CTS severity in surgically managed patients. Further studies examining these factors may better elucidate these factors.

In conclusion, in this study, we demonstrated an association between CTS and TF, which is increased in patients managed surgically for CTS compared with those managed nonsurgically during a 6-month follow-up period. This study used a laterality-specific administrative database to understand these relationships. Additionally, enabled by the number of patients available in such a database, our study is distinct in its use of propensity matching to compare surgically managed patients with CTS with conservatively managed patients. Further studies are required to determine the cause of this association.

Surgeons should be aware of these associations during presurgical and postsurgical evaluations as they might impact patient management. Considering these data, surgeons who detect an early TF may offer more aggressive treatment of TF pathology during CTR. Similarly, surgeons may be more attuned to detecting an early TF during postsurgical assessment with the knowledge of these data.
