# Supplementary material for: Incidence of Trigger Finger in Surgically and Nonsurgically Managed Carpal Tunnel Syndrome
Source: J Hand Surg Glob Online. 2022 Nov 24;5(2):164–8. doi: 10.1016/j.jhsg.2022.10.017 (PMC10039288; doi:10.1016/j.jhsg.2022.10.017)
Supplement: Appendix 1 [file mmc1.docx]

**Appendix 1.** Relevant Current Procedural Terminology Codes and International Classification of Diseases

| **Carpal Tunnel Syndrome Codes** | |
| --- | --- |
| ICD-10: G56 | Carpal Tunnel Syndrome  G56.01 – Right Carpal Tunnel Syndrome  G56.02 – Left Carpal Tunnel Syndrome |
| CPT: 64721 | Carpal Tunnel Release |
| **Trigger Finger Codes** | |
| ICD-10: M65 | Trigger Finger  M65.311 – Right trigger thumb  M65.321 – Right index finger trigger finger  M65.331 – Right middle finger trigger finger  M65.341 – Right ring finger trigger finger  M65.351 – Right small finger trigger finger  M65.312 – Left trigger thumb  M65.322 – Left index finger trigger finger  M65.332 – Left middle finger trigger finger  M65.342 – Left ring finger trigger finger  M65.352 – Left small finger trigger finger |
| CPT: 26055 | A1 pulley release |
| CPT: 20550 | Injection for trigger finger |
